# Torque Teno Virus as a Potential Biomarker for Complications and Survival After Allogeneic Hematopoietic Stem Cell Transplantation

**DOI:** 10.3389/fimmu.2020.00998

**Published:** 2020-05-27

**Authors:** Amandine Pradier, Stavroula Masouridi-Levrat, Carine Bosshard, Carole Dantin, Diem-Lan Vu, Marie-Céline Zanella, Elsa Boely, Caroline Tapparel, Laurent Kaiser, Yves Chalandon, Federico Simonetta, Eddy Roosnek

**Affiliations:** ^1^Division of Hematology, Department of Oncology, Faculty of Medicine, Geneva University Hospitals, Geneva, Switzerland; ^2^Division of Infectious Diseases, Department of Medicine, Faculty of Medicine, Geneva University Hospitals, Geneva, Switzerland; ^3^Translational Research Center for Oncohematology, Department of Internal Medicine Specialties, University of Geneva, Geneva, Switzerland

**Keywords:** TTV, biomarker, CD4, GVHD, HSCT, immunocompetence

## Abstract

Impaired immune reconstitution after allogeneic hematopoietic stem cell transplantation (HSCT) contributes to increased risk of cancer relapse and infection resulting in significant morbidity and mortality. Unfortunately, effective strategies to functionally assess the quality of immune reconstitution are still missing. Quantification of *in vivo* replication of the ubiquitous, non-pathogenic virus Torque Teno Virus (TTV) has been reported in small series as a test to functionally evaluate the quality of post-transplant immune reconstitution. In the present study, we analyzed by quantitative PCR TTV titers in plasma samples from a large cohort of 168 allogeneic HSCT recipients. Our analysis confirms that TTV titers peaked at 100 days post-transplant, followed by progressive normalization thereafter. Negative correlation of TTV titers with T cell absolute numbers during the first year post-transplant points to the restoration of an active anti-TTV immunity. Univariable and multivariable linear regression analysis demonstrated that donor CMV positive serostatus, donor type and immune suppression resulting from GVHD treatment affected the restoration of anti-TTV immunity. Importantly, higher TTV titers at 100 days after transplantation were associated with worse overall survival and higher risk of acute GVHD and infections. Our results provide new insights into the factors affecting the dynamics of TTV replication and indicate that TTV is a potentially useful biomarker to assess immune reconstitution and to predict complications and outcomes of allogeneic HSCT.

## Introduction

Hematopoietic stem cell transplantation (HSCT) is an established treatment for a broad range of hematological disorders. Unfortunately, the pre-transplant conditioning regimen and post-HSCT immunosuppressive therapies induce quantitative and qualitative abnormalities in HSCT recipients' immune system that can result in a severe and often long-lasting immunodeficient status. Impaired immune reconstitution significantly increases the risk of both relapse and transplant related mortality (1–7). Efficient strategies to monitor immune reconstitution are therefore critically important to guide prophylactic and therapeutic interventions. Immunocompetence is difficult to quantify and immune monitoring strategies after allogeneic HSCT vary widely from one center to another (8). The number of T cells is often used as a marker for immune reconstitution (8) but this may be inaccurate because T cells may normalize without restoring immunity (9).

Measuring immunity against ubiquitous, non-pathogenic viruses may represent a test to functionally evaluate post-transplant immune reconstitution. Torque teno virus (TTV), a small non-enveloped anellovirus with a circular single stranded DNA of about 3.8 kb, is highly prevalent in the general population (10) and considered to date to be non-pathogenic with no known associated specific clinical manifestations (11). Plasma levels in immunocompetent individuals are low (12), but HIV infection (13, 14), immune suppression (15–19) or cancer treatment (20) allow the virus to escape immune surveillance and replicate. Importantly, replication of TTV is not affected by antiviral therapies (21). For these reasons several studies assessed the quantification of TTV titers as a precise and straightforward method to measure the patient's immunity. TTV levels after solid organ transplantation (16–19, 21–27) or HSCT (28–34) correlate with the intensity of immunosuppressive treatment and are associated with complications such as rejection (19, 21, 23–25, 35, 36), infections (18, 25–27, 33, 35) or GVHD (30–32, 37).

In this study we prospectively investigated the kinetics of TTV titers and assessed their relationship with clinical parameters and post-transplant reconstitution in a large cohort of allogeneic HSCT recipients. Moreover, we assessed the potential association of TTV titers at day 100 after HSCT with clinical outcomes and post-HSCT complications.

## Methods

### Study Protocol and Patients' Data

One hundred and thirty three adult (≥18 years) patients undergoing a first HSCT for hematological malignancies were enrolled in the study between 2012 and 2015 [91 patients were included in our previous study evaluating TTV titers at time of transplantation (30)]. 3 patients were excluded for early graft failure. In addition, 38 patients transplanted 2 to 9 years before enrolment were recruited. Peripheral blood samples were collected at day 0, 50, 100, 150, 200, 300, 400, 547 and 2 to 9 years post-HSCT (Supplementary Table 1). Patients' characteristics and transplantation related data are presented in Table 1. Ninety one healthy donors [74 of them already included in our previous study (30)] from the Geneva University Hospitals blood transfusion center were also analyzed as a control group. The study was approved by the local ethical committee (n°12-138) and patients and healthy controls (HC) gave their written informed consent.

**Table 1:** Clinical characteristics of HSCT patients.

**Patients and transplant characteristics**		**Patients (*n* = 168)**	
Age, median (IQR)		51	(39–59)
Sex, *n* (%)	F	64	(38)
	M	104	(62)
Diagnosis, *n* (%)	AML	78	(46)
	ALL	17	(10)
	MDS	22	(13)
	MPS	11	(7)
	Lymphoma	12	(7)
	Myeloma	11	(7)
	others	17	(10)
Status at HSCT, *n* (%)	CR	108	(64)
	No CR	60	(36)
DRI, *n* (%)	High/very high	56	(33)
	Low/intermediate	112	(67)
Graft, *n* (%)	PBSC	149	(89)
	BM	19	(11)
Conditioning, *n* (%)	RIC	85	(51)
	MAC	83	(49)
Donor type, *n* (%)	SIB	71	(42)
	MUD	75	(45)
	MMUD	13	(8)
	Haplo	9	(5)
T depletion, *n* (%)	None	30	(18)
	ATG	60	(36)
	pTCD	19	(11)
	ATG+pTCD	50	(30)
	PTCy	9	(5)
CMV status, *n* (%)	D–/R–	47	(28)
	D–/R+	18	(11)
	D+/R–	28	(17)
	D+/R+	75	(45)

*CR, complete remission; DRI, Disease Risk Index; PBSC, peripheral blood stem cell; BM, bone marrow; RIC, reduced-intensity conditioning; MAC, myeloablative conditioning; SIB, identical sibling; MUD, matched unrelated donor; MMUD, mismatched unrelated donor; ATG, anti-thymocyte globulin; pTCD, partial T cell depletion; PTCy, post-transplant Cyclophosphamide*.

### Clinical Protocols

Myeloablative conditioning (MAC) usually consisted of cyclophosphamide (CY 120 mg/kg) in combination with total body irradiation (10–12 Gy) or busulfan (12.8 mg/kg intravenously). Reduced intensity conditioning (RIC) mainly consisted of fludarabine (150 mg/m^2^) associated with low dose busulfan (6.4 mg/kg intravenously) or melphalan (140 mg/m^2^). T cell depletion (TCD) consisted of administration of anti-thymocyte globulin (ATG) and/or “*in vitro*” partial T cell depletion (pTCD) of grafts. ATG (ATG-Thymoglobulin® 7.5 mg/kg or ATG-Fresenius® 25 mg/kg) was part of conditioning for all patients treated with RIC and for patients receiving grafts from an unrelated donor after a MAC. pTCD grafts obtained through *in vitro* incubation with alemtuzumab (Campath® [Genzyme Corporation, Cambridge, MA]), were washed before infusion and administered at day 0, followed on day +1 by an add-back of unmanipulated grafts containing about 100 × 10^6^/kg donor T cells (38). Graft-vs.-host disease prophylaxis mainly consisted of cyclosporine (for 3 months duration in the absence of GVHD in the case of pTCD and for 6 months for T-cell replete graft recipients) in combination with either methotrexate (MTX), in case of MAC, or mycophenolate mofetil (MMF) for patients transplanted after RIC. pTCD graft recipients also received methylprednisolone on days −2 and −1. Patients receiving grafts from haploidentical donors received CY (50 mg/kg) on days 3 and 4 post-HSCT (PTCy). Donor lymphocyte infusions (DLI) at incremental doses starting with 1 × 10^6^ CD3/kg were given at 3 months to all patients who had received pTCD grafts with RIC in the absence of GVHD or independently of TCD to patients with decreasing donor chimerism or in relapse. Acute or chronic GVHD was treated with corticosteroids alone or in combination with mycophenolate mofetil and/or cyclosporine.

### Detection of TTV Viral DNA

Isolation of DNA from frozen plasma was performed using the Nuclisens® Easymag® system (BioMérieux) according to manufacturer's instructions. Plasma were spiked with Canine Distemper Virus (CDV) to control for DNA extraction and serial dilutions of TTV-containing plasmid standard were used for quantification (39). Taqman-based quantitative PCR with primers described by Moen et al. (12) for TTV and Tapparel et al. (40) for CDV was performed. Limit of detection was 25 copies/ml of plasma and the linear amplification ranged from 250 to 2.5 × 10^9^ copies/ml. Patients were considered to control TTV adequately when they had reduced TTV titers below the 90th percentile of the HC group (4 log copies/ml) thereafter.

### Flow Cytometry

Fresh peripheral blood samples underwent red blood cell lysis and cells were stained with monoclonal antibodies specific for the following antigens: CD4 (FITC, clone OKT4, Biolegend), CCR7 (PE, clone 150503, R&D Systems), CD3 (PerCPCy5.5, clone UCHT1, Biolegend), CD8 (APC, clone SK1, Biolegend), CD45 (Alexa Fluor 700, clone HI30, Biolegend), CD56 (Brilliant Violet 421, clone HCD56, Biolegend), CD45RA (Brilliant Violet 510, clone HI100, Biolegend). Data acquired on a Navios flow cytometer (Beckman Coulter) were analyzed with FlowJo^TM^ software (FlowJo LLC). Subsets of CD4 and CD8 where defined according to CD45RA and CCR7 expression as follows: Naïve T cells (T_N_) CD45RA+/CCR7+, central memory T cells (T_CM_) CD45RA–/CCR7+, effector memory T cells (T_EM_) CD45RA–/CCR7– and effector memory re-expressing CD45RA T cells (T_EMRA_) CD45RA+/CCR7–.

### Statistical Analysis

Continuous variables were expressed as median with interquartile range and compared using the non-parametric Mann–Whitney test. Spearman's test was used to determine correlations. Kaplan–Meier's estimates were employed to determine the probability of 2 year overall survival (OS), and progression-free survival (PFS) and differences were determined using the Log-rank Mantel–Cox test. Cox regression was used to examine the independent impact of clinical factors (disease type, disease status, DRI, ATG, pTCD, donor type, and donor CMV serostatus) on OS and PFS. Cumulative incidence estimates of relapse, acute GVHD (grade 2–4) and infections were compared using the Gray test for univariable analysis and the Fine–Gray method for proportional hazard regressions (variables: ATG, pTCD, donor CMV serostatus, disease status, disease type, donor type, and DRI for relapse). Death without relapse and GVHD requiring systemic treatment were considered competing events for relapse. Death and relapse were used as competing events for GVHD. Death, relapse and GVHD requiring systemic treatment were considered competing events for infections. Statistical analysis was performed using Prism version 7 (GraphPad Inc.), R version 3.5.1 [Comprehensive R Archive Network (CRAN) project (http://cran.us.r-project.org)] with R studio Version 1.1.453. *P* < 0.05 were considered significant.

## Results

### TTV Kinetics and Correlation With Immune Reconstitution During the First 2 Years Post-HSCT

**Figure 1:**
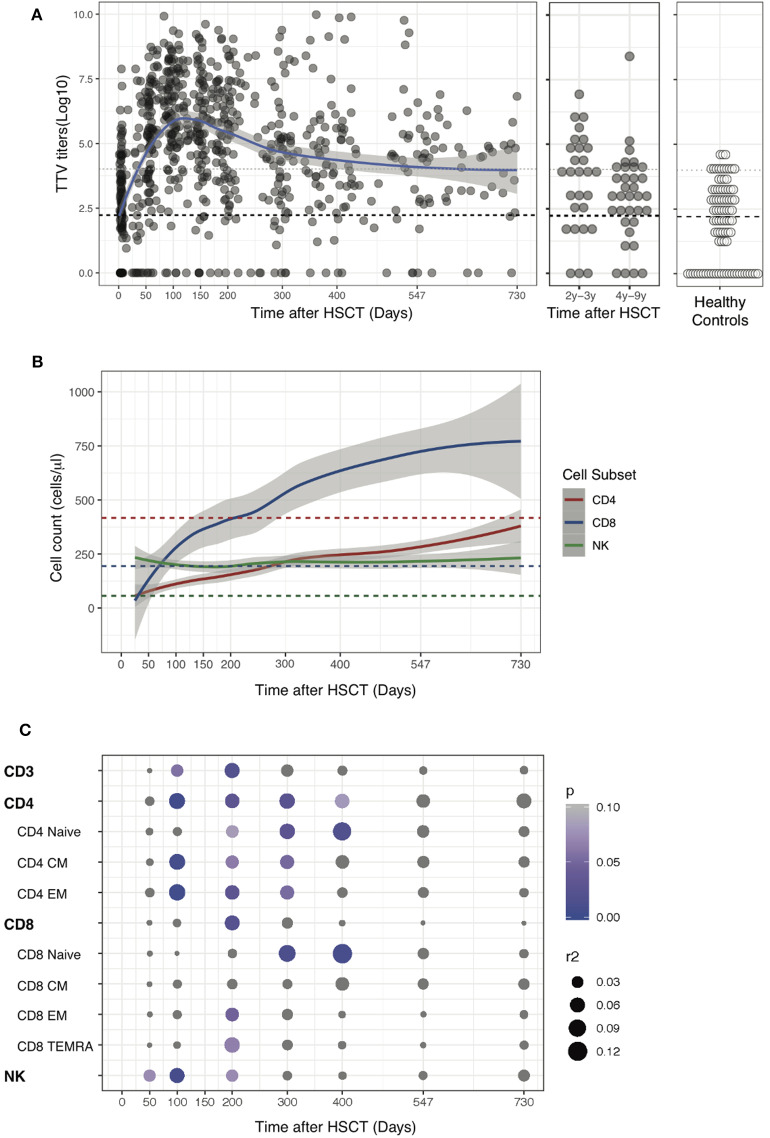
TTV kinetics and correlation with immune reconstitution post-HSCT. **(A)** Data show TTV titers in Log copies/ml of plasma detected in 168 patients up to 9 years post-HSCT or in 91 Healthy Controls (HC). Gray dots represent each sample, blue line represents Loess fit line and the gray area represents the 95% confidence interval (CI) for the regression fit. Median (black dashed line) and 90th percentile (gray dotted lines) of TTV in HC are represented. **(B)** Loess fit lines for the number of NK, CD4, and CD8 in cells/μl are shown post-HSCT. Gray areas represent the 95% CI for the regression fit. **(C)** Correlation between log TTV titer and number of immune cells subsets post-HSCT are presented. The heat map shows Spearman's correlation coefficient and summary of *p*-values for each correlation post-HSCT.

We first measured TTV titers in a cohort of 168 patients at transplant as well as at regular time points thereafter (Figure 1A and Supplementary Table 1). Patients' characteristics and transplantation related data are presented in Table 1. TTV titers in plasma of 91 HC were used as reference (median TTV 2.2 log copies/ml, IQR 0–3.1; Figure 1A). TTV titers post-HSCT varied from undetectable to 10 log copies/ml of plasma. At transplant, the median TTV titer was 2.4 log copies/ml (IQR 0–3.7). TTV titers that had increased slowly over the first 30 days post-HSCT augmented rapidly thereafter to reach a peak at day 100 (median 6.4 log copies/ml, IQR 5.1–7.7). At day 100, only 13% of patients were able to control TTV viremia (< 4 log copies/ml) while at the end of the first year (day 400), 44% of patients had restored sufficient immunity to control TTV viremia. 27/34 (79%) of patients that could be tested after 4 years post-transplant showed TTV levels below 4 log copies/ml (Figure 1A). We next measured the reconstitution of lymphocyte subsets in the same blood samples drawn to determine TTV titers (Figure 1B). NK cells were the first lymphocytes to recover with only 16% of patient having NK cell counts below the lower normal limit at day 50 (lower normal limit = 50 NK/μl; median 155 NK cells/μl, IQR 82–320), while T cells reconstituted more slowly. By day 400, 16% of patients had CD4 T cells above the lower normal limit of 410 CD4/μl (median 192 CD4/μl, IQR 81–332) and 76% of patients had CD8 T cells above lower normal limit (lower normal limit = 190 CD8/μl; 362 CD8/μl, IQR 194–1,045). To investigate the relationship between TTV titers and immune reconstitution we performed a correlation analysis between the number of lymphocyte in each subset and TTV titers (Figure 1C and Supplementary Table 2). At day 100, TTV levels inversely correlated with the number of lymphocytes and more specifically with the number of CD4 T cells and NK cells. Correlation between TTV and CD4 could be observed until day 300 and, from day 300 to 400, TTV titers mainly inversely correlated with CD4 and CD8 naïve subsets characteristic of a thymic rebound (41). No significant correlation with any cell subset was observed before day 100 and after day 400. TTV is known to replicate in hematopoietic cells (29) and TTV titers might therefore also reflect hematopoietic reconstitution in addition to immune-reconstitution. To assess the potential contribution of the hematopoietic reconstitution on TTV titers after transplant, we similarly performed a correlation analysis between total white blood cell (WBC) counts and TTV titers at different time points. We observed an inverse correlation between TTV levels and WBC at day 100 while no correlation was observed at other time points (Supplementary Figure 1).

Collectively, our results confirmed in a large cohort of patients the previously reported kinetics of TTV replication after allogeneic HSCT and uncovered an inverse correlation between TTV titers and immune cell numbers during the first year post-transplant.

### Transplant Characteristics Significantly Affect TTV Titers After Allogeneic HSCT

**Figure 2:**
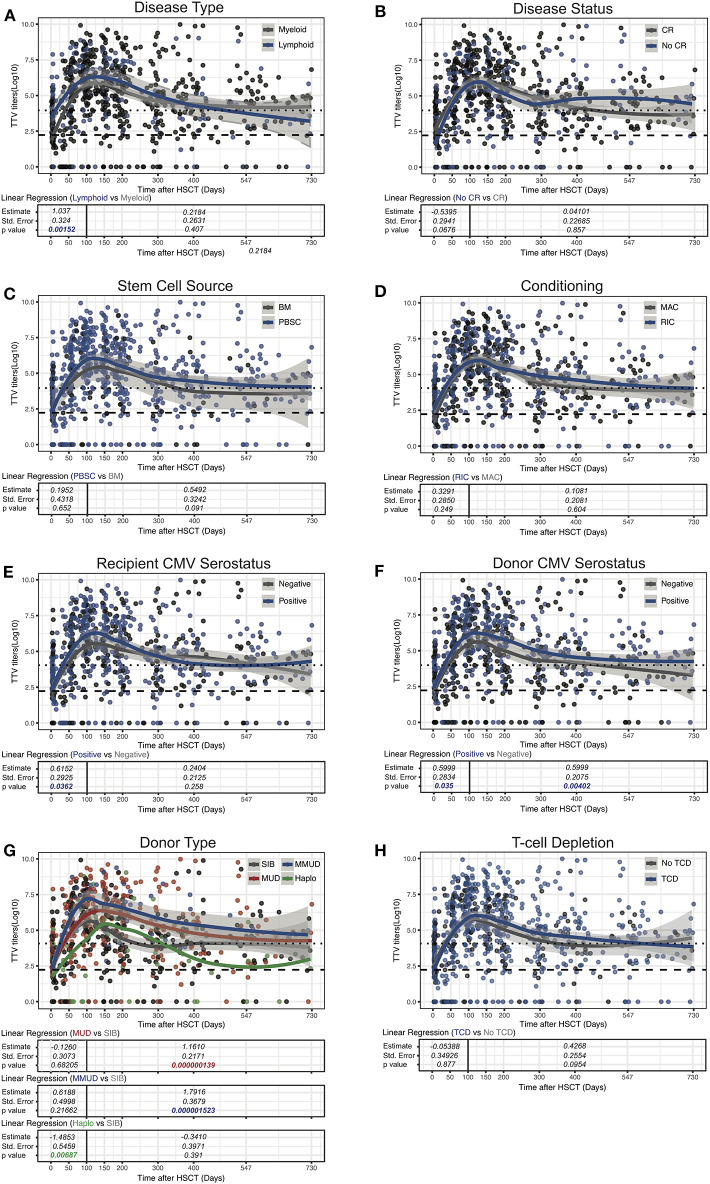
Influence of clinical parameters on TTV titer kinetics in HSCT patients. Data show TTV titers in Log copies/ml of plasma depending on disease Type **(A)**, disease status **(B)**, stem cell source **(C)**, conditioning **(D)**, recipient/donor CMV status **(E,F)**, donor type **(G)**, and T-cell depletion **(H)** are shown. Dots represent each sample, lines represent Loess fit lines for each group and the gray area represents the 95% CI for the regression fit. Estimate, standard error and *p*-values resulting from univariable linear regression analysis are indicated from day 0 to 100 and day 100 to 730. Median (black dashed line) and 90th percentile (gray dotted lines) of TTV in HC are represented.

**Figure 3:**
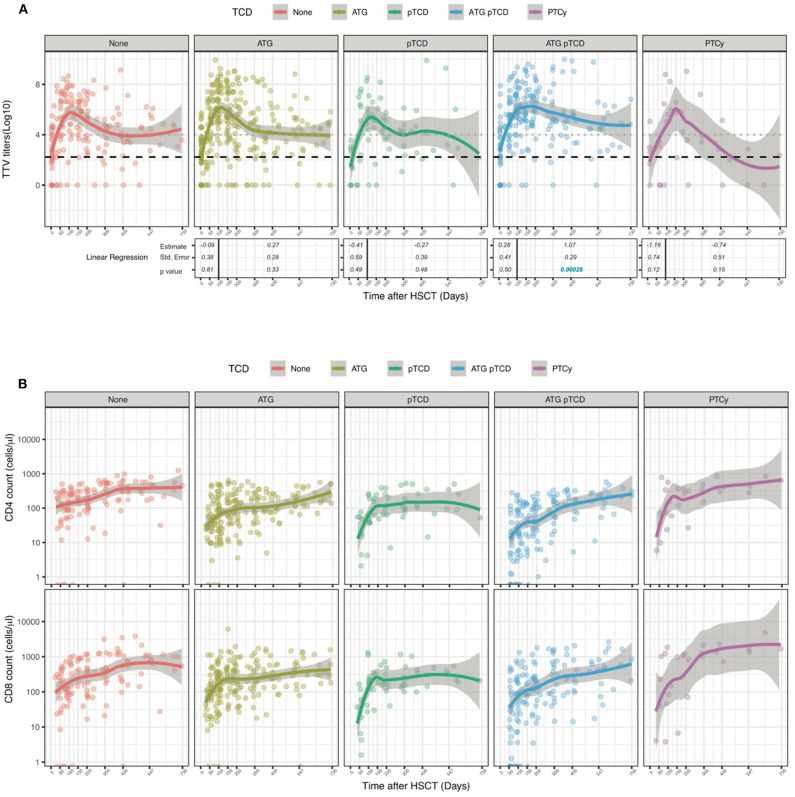
Influence of T cell depletion on TTV titer kinetics in HSCT patients. Data show TTV titers in Log copies/ml of plasma **(A)** and number of CD4 and CD8 cells **(B)** depending on T-cell depletion protocols. Patients received no T depletion (red line, *n* = 30), ATG (yellow line, *n* = 60), pTCD (green line, *n* = 19), ATG plus pTCD (blue line, *n* = 50) or PTCy (purple line, *n* = 9). Dots represent each sample; lines represent Loess fit lines for each group and the gray area represents the 95% CI for the regression fit. Estimate, standard error and *p*-values resulting from univariable linear regression analysis are indicated from day 0 to 100 and day 100 to 730. Median (black dashed line) and 90th percentile (gray dotted lines) of TTV in HC are represented.

We next assessed the impact of clinical factors on TTV titers over the first 2 years after transplantation performing linear regression analysis. Given the biphasic shape of the TTV titers curve post-HSCT (Figure 1A), we separately analyzed time periods before and after day 100. As we and others previously reported (30, 42), patients transplanted for lymphoid malignancies had significantly higher TTV levels at transplant (median 4 log copies/ml, IQR 2.5–5.2) than patients with myeloid malignancies (2.2 log copies/ml, IQR 0–3.2; *p* < 0.0001). This baseline difference significantly impacted TTV kinetics during the first 100 days, but had little impact thereafter (Figure 2A). This difference could also be observed in the multivariable analysis (*p* = 0.0001; Table 2). Factors such as disease status, stem cell source or conditioning had no effect on post-transplant TTV viremia (Figures 2B–D). Recipient and donor positive CMV serostatus was also associated with higher TTV levels in univariable analysis (Figures 2E,F) while multivariable analysis confirmed an association only with CMV positive donor (*p* = 0.0211; Table 2). The strongest association was observed with donor type showing higher TTV viremia for MUD (*p* < 0.0001) and MMUD (*p* < 0.0001) when compared to SIB donors (Figure 2G). Such a difference was confirmed by the multivariable analysis (*p* < 0.0001; Table 2). Patients receiving grafts from haploidentical donors followed by PTCy showed a delay in TTV titers increase early post-HSCT compared to SIB donors (*p* = 0.00687; Figure 2G and Table 2). In these 9 patients, TTV levels remained low over the first 50 days (median 2.9 log copies/ml, IQR 0–3.5 for PTCy vs. 5.7 log copies/ml, IQR 4–7.2 for no TCD; *p* = 0.0004) but rose sharply thereafter reaching their peak at day 150 (5.8 log copies/ml, IQR 4.7–6.6). However, restoration of anti-TTV immunity thereafter was not impaired. We next compared TTV titers in patients in whom T cells were depleted (TCD) by *in vivo* administration of anti-thymocyte globulin (ATG) and/or by partial T cell depletion (pTCD) of the graft and in patients with no TCD. Overall, TTV kinetics in patients receiving TCD were similar to those in no TCD group (Figure 2H). Upon examination of the different TCD methods, only patients receiving ATG together with pTCD grafts exhibited TTV titers higher than no TCD patients (*p* = 0.00029; Figure 3A) although the multivariable analysis failed to confirm these differences (Table 2). The absence of significant difference in TTV kinetics in patients receiving TCD contrasted with the overall delayed T cell reconstitution observed in these patients (Figure 3B and Supplementary Table 3).

**Table 2:** Multivariable analysis of factors influencing TTV levels.

		**Time after HSCT**					
		**Day 0–Day 100**			**Day 100–Day 730**		
**Variable**		**Estimate**	**Std.error**	***p*-value**	**Estimate**	**Std.error**	***p*-value**
**Disease Type**							
	Lymphoid vs. Myeloid	1.16558	0.30327	**0.00015**	0.50480	0.26059	0.05334
**Disease Status**							
	No CR vs. CR	−0.86492	0.30019	**0.00424**	0.39503	0.23827	0.09802
**Stem Cell Source**							
	PBSC vs. BM	−0.16618	0.47058	0.72422	0.48762	0.38754	0.20894
**Conditioning**							
	RIC vs. MAC	0.17429	0.30546	0.56871	0.30143	0.24695	0.22286
**CMV Serostatus**							
	Recipient Positive vs. Negative	0.05642	0.30914	0.85530	0.05890	0.23663	0.80354
	Donor Positive vs. Negative	0.32628	0.29764	0.27384	0.53619	0.23170	**0.02110**
**Donor Type**							
	MUD vs. SIB	−0.55652	0.32449	0.08735	1.06928	0.25243	**0.00003**
	MMUD vs. SIB	0.11550	0.52532	0.82613	1.89318	0.44265	**0.00002**
	Haploidentical vs. SIB	−2.63122	0.80736	**0.00124**	−0.02353	0.73214	0.97437
**T-cell Depletion**							
	ATG	0.09700	0.40319	0.81004	−0.36422	0.33804	0.28185
	pTCD	−0.15317	0.58536	0.79376	0.61423	0.43423	0.15789
	ATG pTCD	0.39477	0.44782	0.37871	0.39199	0.33908	0.24827
	PTCy	0.66309	0.96078	0.49062	−0.32707	0.84003	0.69719
**GVHD before sampling**							
	Previous GVHD vs. No GVHD	2.59324	0.32105	**<0.00001**	0.51264	0.22753	**0.02472**

*CR, complete remission; PBSC, peripheral blood stem cell; BM, bone marrow; RIC, reduced-intensity conditioning; MAC, myeloablative conditioning; SIB, identical sibling; MUD, matched unrelated donor; MMUD, mismatched unrelated donor; ATG, anti-thymocyte globulin; pTCD, partial T cell depletion; PTCy, post-transplant Cyclophosphamide; Statistically significant results are highlighted in bold*.

Collectively, these results demonstrated an impact of disease type and donor characteristics on TTV kinetics after allogeneic HSCT.

### GVHD and Its Treatment Significantly Impact TTV Titers

**Figure 4:**
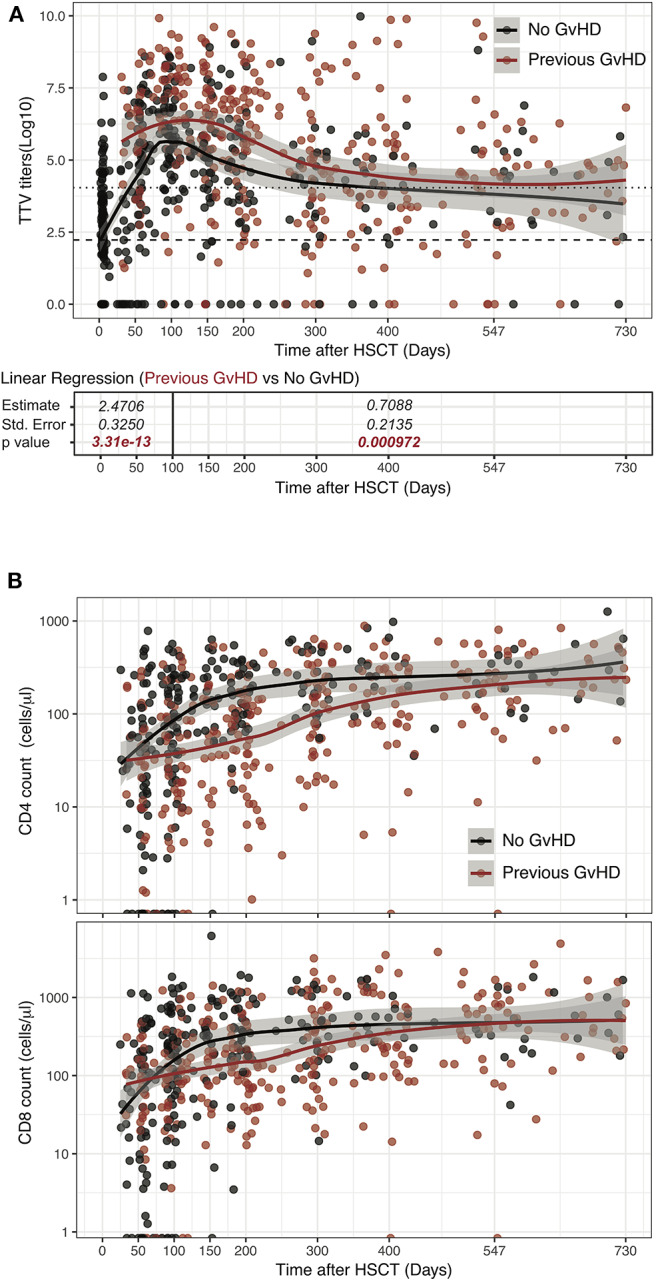
Influence of GVHD occurrence on TTV titer kinetics in HSCT patients. Log TTV copies/ml of plasma **(A)** and number of CD4 and CD8 cells **(B)** are represented. Patients' samples obtained after onset of GVHD requiring immunosuppression are depicted in red and compared to samples obtained from patients without GVHD (black dots). Lines represent Loess fit lines for each group and the gray area represents the 95% CI for the regression fit. Estimate, standard error and *p*-values resulting from linear regression analysis are indicated from day 0 to 100 and day 100 to 730. Median (black dashed line) and 90th percentile (gray dotted lines) of TTV in HC are represented.

To examine the effect of GVHD and its treatment on anti-TTV immunity, we stratified the patients based on the development of acute or chronic GVHD requiring systemic immune-suppression in the first 2 years post-HSCT. 83 patients suffered from GVHD requiring systemic treatment which occurred at a median day 50 (IQR 20–137). Patients received corticosteroids (82 patients), calcineurin inhibitors (75 patients), MMF (31 patients), photopheresis (16 patients), or basiliximab (4 patients). Univariable linear regression analysis demonstrated that after the onset of GVHD, patients had significantly higher TTV levels than patients without GVHD (d0-100: *p* < 0.00001 and d100-730: *p* < 0.001; Figure 4A). TTV titers were significantly higher at day 100 in patients affected by GVHD (median 6.9 log copies/ml, IQR 5.4–7.9) compared with patients not experiencing the complication (5.6 log copies/ml, IQR 4.4–6.6; *p* = 0.013). This association was confirmed in the multivariable analysis (Table 2). It is notable that during the first year post-HSCT the number of CD4 and CD8 T-cells in patients with GVHD remained significantly lower than in patients without GVHD (Figure 4B and Supplementary Table 4).

Twenty-three patients relapsed over the study period and no differences in TTV titers could be observed between these patients and patients who did not relapse (Supplementary Figure 2). However, due to the great heterogeneity in underlying diseases and relapse treatments it is difficult to draw any solid conclusions.

These data indicate that post-transplant complications, namely GVHD, and immunosuppressive drugs employed for its treatment have an impact on TTV replication kinetics.

### Higher TTV Titers at Day 100 Are Associated With Worse Overall Survival and Increased Risk of GVHD and Infections

**Figure 5:**
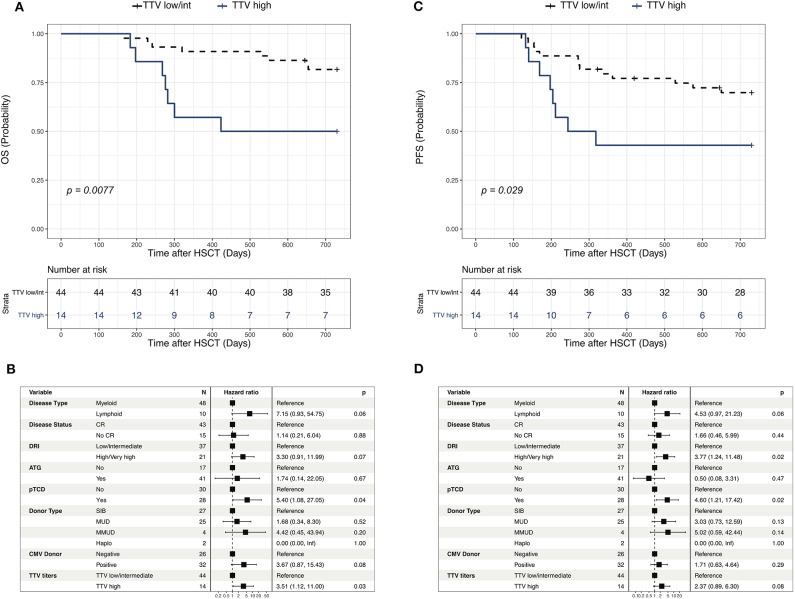
Outcome of patients according to TTV level at d 100 post-HSCT. **(A,C)** Kaplan-Meier curves showing the impact of TTV level at day 100 on overall survival (OS, **A**) and Progression free survival (PFS, **C**). Comparison between high (upper quartile) and low/intermediate (first to third quartiles) TTV groups was performed using Log-rank test. **(B,D)** Multivariable Cox regression examining the independent impact of clinical factors on OS **(B)** and PFS **(D)**. Hazard ratios (HR) along with their 95% CI are presented. High TTV levels were defined as values above the 75th percentile of the TTV titers at day 100 (>6.705 log copies/ml).

**Figure 6:**
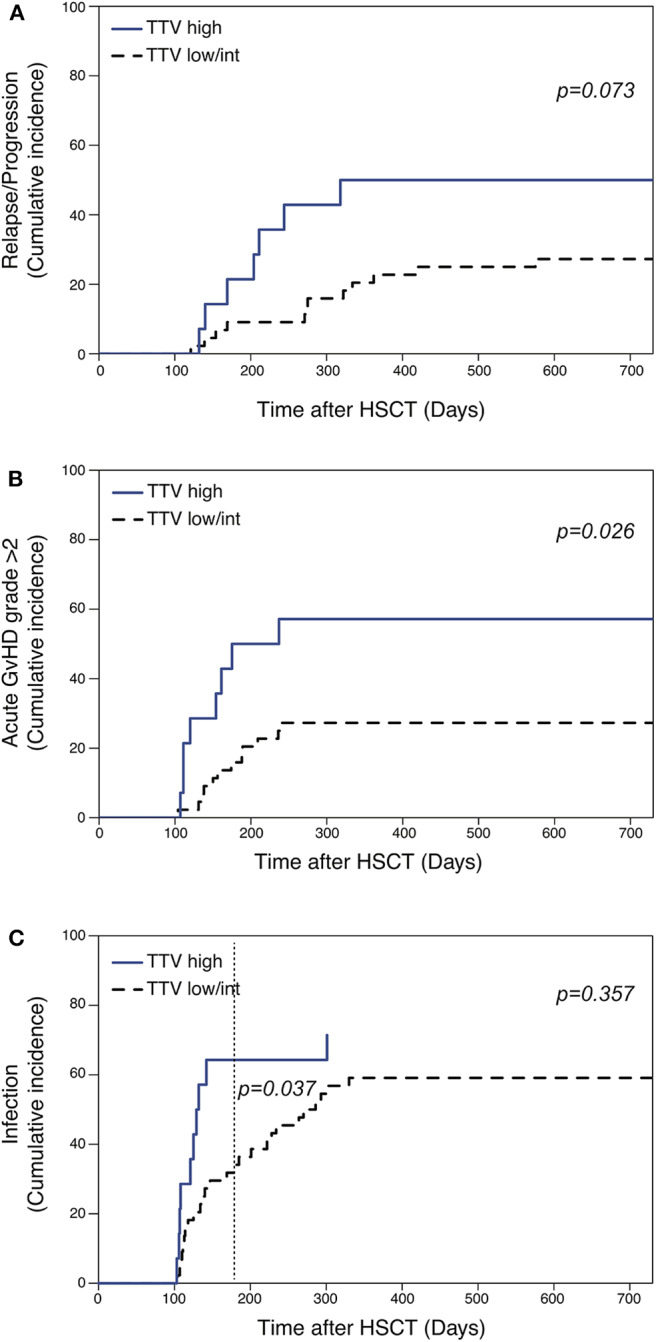
Occurrence of post-HSCT complications according to TTV level at d 100 post-HSCT. Impact of TTV levels at day 100 on relapse **(A)**, acute GVHD (grade ≥2) **(B)** or infections **(C)** cumulative incidence. Competing risk factors were defined as death, GVHD requiring systemic therapy (for relapse and infections) and relapse (for GVHD and infections). High TTV levels were defined as values above the 75th percentile of the TTV titers at day 100 (>6.705 log copies/ml). Gray test was used for comparison between groups.

We next performed a landmark analysis to assess the potential association between TTV titers measured at day 100 and outcome after allogeneic HSCT. The landmark analysis was restricted to 58 patients that were alive without evidence of disease relapse and/or GVHD requiring systemic treatment at day 100. Patients displaying day 100 TTV titers in the upper quartile (threshold of 6.705 log copies/ml) displayed a significantly worse 2 year OS (50%, 95%CI 30–84%) compared to patients with lower TTV titers (82, 95% CI 71–94%; Figure 5A). Such a difference was confirmed in a multivariable analysis performed taking into account transplant and disease characteristics (HR 3.5, 95% CI 1.1–11; *p* = 0.03; Figure 5B). Univariable analysis similarly showed worse PFS in patients with high TTV titers at day 100 (43, 95% CI 23–78%) compared to patients with lower TTV titers (70, 95%CI 57–85%; Figure 5C), although the multivariable analysis failed to confirm this difference (Figure 5D). To gain further insights into reasons behind the associations between TTV titers and transplantation outcomes, we assessed the relationship between TTV titers at day 100 and occurrence of post-HSCT complications. Using the cut-off defined above, we performed cumulative incidence analysis for relapse, acute GVHD (grade 2–4) and infections (Figure 6). We observed a tendency not reaching statistical significance toward higher relapse rates in patients with higher TTV titers (*p* = 0.073; Figure 6A). Patients with high TTV titers had higher rates of acute GVHD (*p* = 0.026; Figure 6B), a result confirmed in a multivariable analysis (HR ± SE: 2.940 ± 0.522, *p* = 0.039) performed taking into account the abovementioned factors (disease type and status, donor type and CMV serostatus, TCD). No significant difference in 2 year cumulative incidence of infections was observed between patients groups stratified based on TTV titers. As the immune reconstitution status at day 100 is more likely to affect the infection risk at short term, we assessed the cumulative incidence of infections in the months following the measurement. Patients displaying higher TTV titers at day 100 had higher rates of infection at 6 months post-transplant (*p* = 0.025; Figure 6C) a result confirmed by the multivariable analysis (2.649 ± 0.466; *p* = 0.037).

Collectively, these results demonstrated the association between TTV titers measured at day 100 and post-transplant complications and overall survival.

## Discussion

Treatment induced immune deficiency may impair the curative role of allogeneic HSCT. Clinical parameters, the type of conditioning and the patient's state before transplantation impact reconstitution of the immune system. The interaction of these numerous parameters is so complex that it becomes virtually impossible to predict the patient's immunocompetence at a given stage after HCST. Being able to assess patient's immunity is important because post-transplant interventions and therapies could be adapted accordingly. Unfortunately, no objective parameter reflecting the level of immune reconstitution has been established to date.

Over the past decade several groups investigated the kinetics of TTV after transplantation and asked the question whether TTV titers could serve as a comprehensive marker of post-transplant immunity. Data from solid organ transplantation where TTV titers correlate with the strength of IS (16–19) and predict infections (18, 25–27, 35) as well as rejections (19, 21, 23–25, 35, 36) are encouraging. Unfortunately, the situation after HSCT seems to be more complex probably owed to the vast heterogeneity of patients and treatment modalities.

Here we report the results of a cohort of 168 patients transplanted in our center. As previously reported (31, 32, 34, 37, 42), we found that TTV titers increased rapidly after transplantation peaking at around 100 days post-HSCT, which corresponds to the time that IS is tapered. Moreover, donor CMV positive serostatus, donor type and immune suppression resulting from GVHD requiring systemic immunosuppression influenced the restoration of anti-TTV immunity. These results are in agreement with the inverse correlation we (Figure 1C) and others (42) observed between immune reconstitution and viral titers. However, our analysis of subgroups of patients receiving *in vivo* and/or *ex vivo* T cell depletion failed to reveal any impact of TCD strategies on TTV titers. This apparently surprising result might be related to the lack of statistical power of the subgroup analysis and/or from the insufficient ability of total CD4 and CD8 T cell counts to reflect the functional immune status of HSCT recipients (9).

Hematopoietic cells are thought to be the main replication competent cells (29, 43–46). TTV titers might therefore reflect both the hematological and immunological reconstitution. Our correlation analysis between total white blood cells, a common measure of hematological reconstitution, and TTV titers failed to demonstrate any positive correlation between WBC counts and TTV titers at early time points and revealed only a negative correlation at day 100. This result suggests that, at least after hematopoietic engraftment, TTV titers are mainly influenced by the degree of immune-reconstitution.

Studies in patients after HSCT have not yet revealed whether patient or treatment related variables might impact long-term anti-TTV immunity (30–32, 34, 42) and, more importantly, how the control of TTV replication might affect transplantation outcomes. This could be simply due to the fact that the patients in these studies were mainly monitored only during the early follow-up period when only very few patients manage to control the virus. In 2017, Wohlfarth et al. have looked beyond this early phase in a first prospective longitudinal study. We confirm their findings with respect to the impact of GVHD and/or its treatment that increase TTV titers. In addition, they found that increasing TTV titers were associated with CMV/EBV reactivation but concluded that, owed to presence of the many transplant-related confounding factors such as conditioning, GVHD and IS, they could not be predictive of other immune-related clinical complications. More recently, in a retrospective study, Schmitz et al. (34) also investigated TTV as an early (before day 50) prognostic marker after HSCT but failed to show any association. Using multivariable analyses and cumulative incidences taking into account competing events, our study overcomes some limitations encountered in previous studies and shows that high TTV titers at day 100 may be indicative of OS and flag an increased risk of GVHD and infection and possibly of relapse. Day 100 appears to be a suitable time point for TTV titers to be used as a prognostic biomarker. Firstly, because it is the time when TTV replication reaches its peak and starts to be affected by the degree of immune reconstitution rather than by pre-transplant factors. Secondly, because day 100 represents a time point at which critical decisions are taken regarding immunosuppressive treatment duration, antimicrobial prophylaxis and immune interventions. If confirmed, our results might pave the way to clinical trials assessing the feasibility of tailoring the prescription of immune-suppressive drugs on the TTV titers. A similar approach is currently under investigation in clinical trials in solid organ transplantation recipients (NCT04198506). Such a strategy might allow to more efficiently prevent GvHD and to limit the administration of unnecessarily high, and potentially toxic, levels of immunosuppressive treatments.

The current study, as well as most of those reported in the literature, employed an in-house assay. Future multicenter clinical trials evaluating the potential clinical use of TTV titers as a biomarker of functional immune-reconstitution after allogeneic HSCT would greatly benefit from the use of standardized methods of TTV quantification. Since the beginning of our study, a commercial kit for TTV quantification became available (TTV R-gene® kit; ARGENE®, bioMérieux, France) and its use in future clinical trials could enable higher comparability between laboratories.

Our study has several limitations. First, the size of cohort we studied for our landmark analysis is small and very heterogeneous with respect to the patient's disease, state and treatment, which certainly introduces many confounders, several of which may have remained unnoticed. Because of the limited size of our cohort, our proof-of-concept analysis of the association between TTV titers at day 100 and clinical outcomes was based on an arbitrarily defined cutoff (upper quartile). Moreover, this cutoff was tested in the same cohort in whom it was established without external validation in an independent cohort. Finally, the number of patients experiencing relapse is too limited to draw any solid conclusion on the relationship between viral titers and antitumor immunity. Nevertheless, we believe that our data based on a simple laboratory test warrant further investigation in prospective multicenter clinical trials to assess TTV titers as a marker to predict complications and outcome of allogeneic HSCT.

## Data Availability Statement

The datasets generated for this study are available on request to the corresponding author.

## Ethics Statement

The studies involving human participants were reviewed and approved by Commission Cantonale d'Ethique de la Recherche sur l'être humain de Genève. The patients/participants provided their written informed consent to participate in this study.

## Author Contributions

ER designed the study. AP and CB performed the experiments. AP, CD, D-LV, EB, M-CZ, and SM-L collected the clinical data. AP, ER, and FS interpreted the data and wrote the manuscript. AP and FS analyzed the data, performed statistical analysis, and prepared figures. LK, CT, SM-L, and YC provided overall guidance and critically revised the manuscript. All authors have approved the final version of the manuscript.

## Conflict of Interest

The authors declare that the research was conducted in the absence of any commercial or financial relationships that could be construed as a potential conflict of interest.

## Supplementary Material

Supplementary Figure 1 Correlation of TTV titers with total white blood cell (WBC) counts. Correlation of TTV titers at different time points with WBC counts. Correlations evaluated using a Spearman rank correlation coefficient test are indicated. *P* < 0.05 was considered statistically significant.

Supplementary Figure 2 Influence of relapse occurrence on TTV titer kinetics in HSCT patients. Log TTV copies/ml of plasma are represented. Patients' samples obtained after disease relapse are depicted in blue and samples obtained from patients without relapse in black. Lines represent Loess fit lines for each group and the gray area represents the 95% CI for the regression fit. Median (black dashed line) and 90th percentile (gray dotted lines) of TTV in HC are represented.

Supplementary Table 1 Sample collection schedule and TTV titers in the study population.

Supplementary Table 2 Correlation between TTV titers and immune reconstitution.

Supplementary Table 3 Number of CD4 and CD8 post-HSCT according to T cell depletion.

Supplementary Table 4 Number of CD4 and CD8 post-HSCT according to GVHD occurrence.
