# Supplementary material for: Torque Teno Virus as a Potential Biomarker for Complications and Survival After Allogeneic Hematopoietic Stem Cell Transplantation
Source: Front Immunol. 2020 May 27;11:998. doi: 10.3389/fimmu.2020.00998 (PMC7267041; doi:10.3389/fimmu.2020.00998)
Supplement: Supplementary Table 1 — Sample collection schedule and TTV titers in the study population. [file Data_Sheet_1.docx]

# **Supplementary Material**

**Supplementary Table 1.** **Sample collection schedule and TTV titers in the study population.**

| **Time points** | **number of patients** | **Planed schedule in days** | **Median days (IQR)** | **Median TTV in log copies/ml (IQR)** |
| --- | --- | --- | --- | --- |
| d0 | 130 | 0 (+15) | 5 (4-6) | 2.4 (0-3.7) |
| d50 | 124 | 50 (±25) | 56 (45-60) | 5.1 (3.3-6.6) |
| d100 | 115 | 100 (±25) | 97 (91-105) | 6.4 (5.1-7.7) |
| d150 | 103 | 150 (±25) | 149 (144-156) | 5.8 (4.6-6.9) |
| d200 | 95 | 200 (±25) | 199 (188-206) | 5.6 (3.7-7.1) |
| d300 | 81 | 300 (±50) | 297 (286-308) | 4.7 (3.1-5.6) |
| d400 | 64 | 400 (±50) | 391 (366-412) | 4.3 (3.4-5.6) |
| d547 | 58 | 547 (±90) | 548 (518-568) | 4.4 (2.8-5.4) |
| d730 | 46 | 730 (±90) | 726 (679-748) | 3.5 (2.6-4.8) |
| y3 | 28 | 1095 (±180) | 1077 ( 957-1101) | 3.9 (2.0-4.9) |
| y4-9 | 34 | 1278-3367 | 1816 (1461-2156) | 3.0 (2.0-4.0) |

**Supplementary Table 2. Correlation between TTV titers and immune reconstitution.**

**
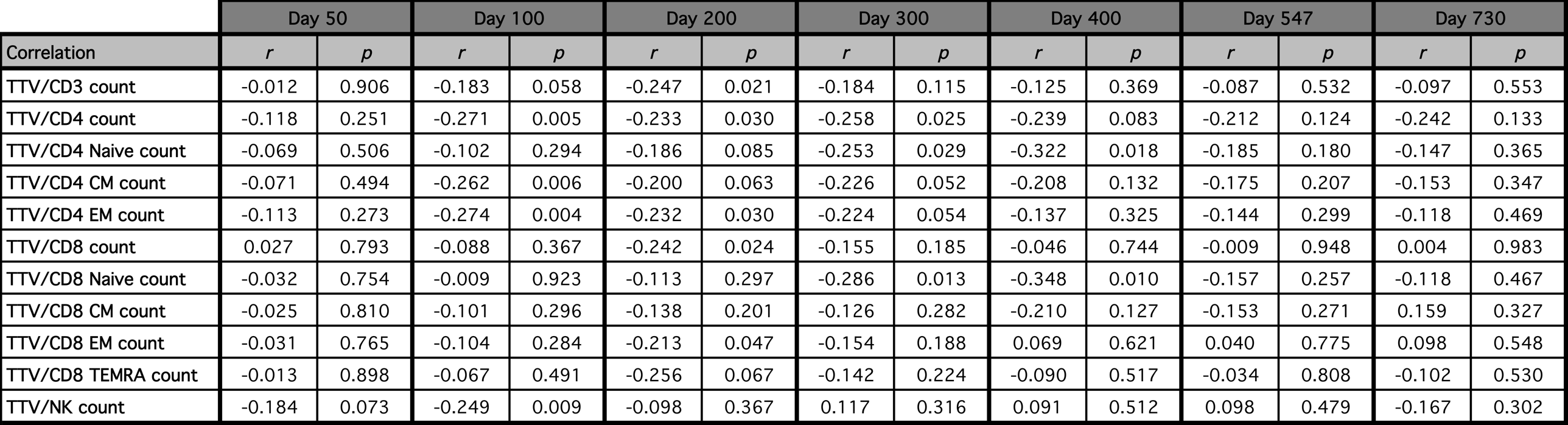
**

**Supplementary Table 3. Number of CD4 and CD8 post-HSCT according to T cell depletion.**





**Supplementary Table 4. Number of CD4 and CD8 post-HSCT according to GVHD occurrence.**

| **CD4** | **GVHD** | | **no GVHD** | |  |
| --- | --- | --- | --- | --- | --- |
|  | median (IQR) | | median (IQR) | | p value |
| **d50** | 27 | (8-66) | 29 | (5-95) | 0.7617 |
| **d100** | 31 | (11-93) | 86 | (26-233) | **0.0247** |
| **d150** | 61 | (25-143) | 147 | (69-276) | **0.0090** |
| **d200** | 68 | (25-137) | 172 | (98-307) | **<0.0001** |
| **d300** | 127 | (40-275) | 203 | (105-462) | **0.0162** |
| **d400** | 176 | (79-309) | 306 | (196-498) | **0.0226** |
| **d547** | 240 | (118-429) | 248 | (115-372) | >0.9999 |
| **d730** | 307 | (185-444) | 290 | (209-600) | 0.6758 |
| **CD8** | **GVHD** | | **no GVHD** | |  |
|  | median (IQR) | | median (IQR) | | p value |
| **d50** | 71 | (28-1595) | 41 | (4-205) | 0.4504 |
| **d100** | 113 | (43-186) | 186 | (31-371) | 0.1604 |
| **d150** | 165 | (81-467) | 323 | (92-611) | 0.4381 |
| **d200** | 128 | (69-240) | 374 | (172-828) | **0.0004** |
| **d300** | 219 | (83-706) | 443 | (156-949) | 0.1804 |
| **d400** | 269 | (146-1022) | 568 | (304-1045) | 0.1443 |
| **d547** | 611 | (287-1014) | 394 | (218-584) | 0.3578 |
| **d730** | 380 | (176-885) | 364 | (195-942) | 0.9137 |
